# Integrating Numerical Data with AI-Based Image Processing Techniques to Improve the Diagnostic Accuracy of Detecting Dental Caries in Panoramic Radiographs

**DOI:** 10.3390/diagnostics15243167

**Published:** 2025-12-12

**Authors:** Bengü Başarı, Nuran Ulusoy, Kamil Dimililer

**Affiliations:** 1 Department of Restorative Dentistry, Faculty of Dentistry, Near East University, N. Cyprus/Mersin 10, Nicosia 99138, Türkiye; nuran.ulusoy@neu.edu.tr; 2 Department of Electrical and Electronic Engineering, Applied Artificial Intelligence Research Center (AAIRC), Research Center for Science, Technology and Engineering (BILTEM), Near East University, N. Cyprus/Mersin 10, Nicosia 99138, Türkiye; kamil.dimililer@neu.edu.tr

**Keywords:** dental caries, image processing techniques, artificial intelligence (AI), panoramic radiography, numerical image data

## Abstract

**Background/Objectives**: Dental caries is among the most common oral health problems, resulting from demineralization of dental hard tissues in acidic environments. Early diagnosis is essential to prevent severe tissue destruction, systemic complications and costly treatments. Conventional visual interpretation of panoramic radiographs, though widely used, remains subjective and variable. This study evaluated the effectiveness of image processing techniques and artificial intelligence (AI)-assisted models for automated detection and classification of dental caries on panoramic radiographs, emphasizing numerical image data analysis. **Methods**: From 1084 panoramic radiographs, 405 were selected and classified into interproximal, occlusal and secondary caries groups. Each was segmented and one representative region was analyzed using the image data representation method. Numerical descriptors—brightness, contrast, entropy and histogram parameters—were extracted and evaluated with several machine learning algorithms. **Results:** Among tested models, the Decision Tree algorithm achieved the highest classification accuracy (0.988 at the 0.2 train-test ratio), showing superior and consistent results across caries types. Random Forest also demonstrated strong performance with limited training data, while Gaussian Naïve Bayes, KNN and RBFNN were less effective. **Conclusions**: The integration of numerical image features with AI-based models demonstrated high diagnostic accuracy and clinical interpretability, particularly with Decision Tree algorithm. These results highlight the potential of AI-assisted analysis of panoramic radiographs to enhance diagnostic reliability, reduce subjectivity and support more effective treatment planning. Further multicentre studies with larger and more diverse datasets are recommended to validate generalizability.

## 1. Introduction

Dental caries, resulting from the demineralization of hard dental tissues in an acidic environment, are among the most prevalent oral health issues worldwide, impairing both the structural integrity and functional capacity of teeth [1]. Their high prevalence in both developed and developing nations poses a significant public health concern. If left undiagnosed in the early stages, caries can progress to advanced levels, causing extensive tissue destruction, systemic complications and the need for costly treatment interventions. For this reason, prompt and accurate detection is regarded as a key factor in ensuring successful treatment outcomes and improving patient satisfaction [2]. Panoramic radiography is one of the most frequently employed imaging modalities for caries detection, offering the advantage of visualizing the entire oral cavity in a single exposure with minimal radiation dosage [3]. Despite these advantages, conventional evaluation of panoramic radiographs relies heavily on the clinician’s visual interpretation, a process vulnerable to subjective errors due to factors such as reduced attention over time, variability in clinical experience and fatigue [4]. Furthermore, differences in image quality and anatomical variations among patients make it difficult to maintain diagnostic consistency [5]. Previously, several diagnostic methods were used for the diagnosis of dental caries [6] including visual, tactile and radiographic examination, image processing and segmentation, machine learning algorithms and image data representation methods.

In recent years, artificial intelligence (AI) and advanced image processing techniques have emerged as promising tools for faster, more objective and more reliable detection of dental caries. AI algorithms can apply a range of high-precision image enhancement and analysis methods, including contrast optimization, noise suppression, edge detection, morphological filtering and lesion segmentation [7]. These processes enhance the visibility of carious lesions, enabling earlier diagnosis [8]. Additionally, numerical data derived during image processing—such as pixel intensity distributions, edge sharpness metrics, texture descriptors and histogram-based parameters can serve as valuable inputs for AI-based classification models, thereby substantially improving their diagnostic performance [9]. With rapid advancements in deep learning and sophisticated image analysis approaches, the potential for panoramic radiographs to be used more effectively in caries detection has grown significantly [10]. Recent studies emphasize that dividing panoramic radiographs into smaller, more consistent segments prior to analysis can enhance model learning capacity and overall performance [11]. Post-segmentation application of advanced enhancement techniques, such as contrast boosting, noise reduction and edge refinement, further contributes to the creation of rich and high-quality datasets, improving both the generalizability and accuracy of AI models [12].

The initial step in digital image processing is image acquisition. This process begins with the conversion of real-world physical objects into a digital format using appropriate imaging devices. Digital image processing involves the transfer of sensor-acquired data into a computer environment, the use of various processing techniques and the visualization of the resulting image through an output device. The hardware and software used for image acquisition may vary depending on the nature of the image source. Commonly employed imaging tools include CCD cameras, ultrasound systems, infrared sensors, magnetic resonance imaging (MRI) systems, other sorts of X-ray devices and satellite-based technologies. The output from the sensors is typically an analogue voltage signal that varies continuously depending on the object being observed. In order to convert this analogue data into a digital image, two essential steps are required: sampling and quantization. Sampling refers to the digitization of the spatial (coordinate) components of the image, while quantization involves the digitization of the amplitude (intensity) values. To generate a digital image, both the spatial coordinates and the amplitude of the image function must be discretized [13].

Computer vision-based systems provide a more reliable alternative to traditional methods by processing dental images with high accuracy. These technologies enable the early detection of caries by capturing structural changes that are not visible to the naked eye. For instance, object recognition algorithms can identify areas of demineralization, which are often the earliest indicators of caries development. Semantic segmentation techniques further enhance this process by dividing the image into distinct regions, allowing for a more detailed examination of the tooth surface and surrounding tissues. This level of detailed analysis is particularly valuable for identifying caries that develop in hard-to-detect areas, especially in restorations such as between teeth or beneath. In addition, AI-assisted diagnostic approaches can evaluate patterns of caries progression, providing clinicians with predictions about the future course of the disease and supporting the planning of appropriate treatment strategies. Integrating such advanced image processing techniques into routine dental practice will allow for faster and more effective interventions, thereby reducing the prevalence of advanced caries cases and improving overall oral health outcomes [14]. Machine learning techniques are commonly categorized into four main groups: supervised, unsupervised, semi-supervised and reinforcement learning [15]. The growing interest further highlights the importance of investigating ML in contemporary research.

The effectiveness of any ML-based solution depends on both the distinctive features of the data and the suitability of the algorithms applied. Within this field, a variety of approaches—such as classification, regression, clustering, dimensionality reduction, feature engineering, association rule mining and reinforcement learning—are utilized to construct robust data-driven systems [16,17]. Deep learning, derived from artificial neural networks, has also become a dominant subfield, offering advanced methods for analyzing complex datasets [18]. Deep learning, which stems from the principles of artificial neural networks, has evolved into a prominent field by providing sophisticated techniques for handling complex forms of data. Nevertheless, determining the most suitable learning approach for a particular task remains difficult. Different algorithms are designed with distinct goals in mind and even models within the same category can yield varying results depending on the characteristics of the dataset being used [19].

This study focuses on highlighting the potential of combining advanced techniques of image processing with artificial intelligence to enhance the accuracy and efficiency of dental caries detection on panoramic radiographs. Unlike previous research, our work uniquely focuses on the extraction and analysis of numerical image data, which has not been explored in prior studies. The primary aim of this study was to investigate whether numerical image features could serve as objective indicators for caries detection, thereby reducing diagnostic subjectivity. The secondary aim was to compare the diagnostic performance of multiple machine learning algorithms to determine which model offers the greatest clinical utility, considering various train-test ratios.

Based on the clinical challenges associated with detecting different types of dental caries on panoramic radiographs and the potential advantages of artificial intelligence–based diagnostic systems, the following hypotheses were formulated:

**H01:**  *Image processing techniques with numerical parameters, especially for visually difficult to distinguish interproximal caries, occlusal caries and secondary caries will demonstrate high performance.*

**H02:**  *Machine learning algorithms will show high accuracy in detecting dental caries.*

**H03:**  *Machine learning algorithms will show no difference in accuracy and sensitivity between interproximal caries, occlusal caries and secondary caries.*

## 2. Materials and Methods

In this study, panoramic radiographs obtained from patients who applied to our faculty between 2024 and 2025 were retrospectively analyzed. The radiographs were retrieved from the faculty archives. Ethical approval (NEU/2025/135-1986) was granted by the Near East University Scientific Research Ethics Committee (Decision Date and Number: 26 June 2025/135, ensuring compliance with ethical standards and participant protection of Declaration of Helsinki.

### 2.1. Evaluation and Categorization of Panoramic Radiographs

A total of 1084 panoramic radiographs from individuals aged 18 or above were examined with the Orthophos SL 3D and Orthophos XG units (Dentsply Sirona, Bensheim, Germany) at 60–90 kVp and 3–16 mA. For consistency and reliability, certain images were excluded because of poor quality, presence of artifacts, or incomplete anatomical structures. Artifacts were defined as artificial distortions not representing actual anatomical structures, typically caused by patient movement, metallic restorations, technical limitations, or improper positioning. After exclusion of inadequate images, only radiographs meeting the required quality standards were included in the analysis. As the dental faculty hospital serves a wide and diverse patient population due to its international structure, the dataset reflects a multi-ethnic clinical group rather than a single racial background.

In the initial evaluation phase, carious lesions visible on the panoramic radiographs were independently assessed by two experienced dentists. Following this step, each image was cropped to standardized dimensions in order to include only the teeth and root structures, deliberately excluding other anatomical areas. The cropping process ensured uniformity across the dataset. Each selected radiograph was divided into 15–100 image segments and one representative segment per radiograph was chosen for further analysis. Data was extracted using the image data representation method, resulting in numerical descriptors.

For the purposes of the study, the radiographs were categorized into three caries groups: interproximal caries, occlusal caries and secondary caries. From the initial dataset, 135 radiographs were selected for each group (a total of 405 radiographs) to maintain balance among the categories for subsequent analysis.

### 2.2. Image Processing and Segmentation

In this study, panoramic radiographs were categorized into three groups: interproximal caries, occlusal caries and secondary caries. For each category, 135 radiographs were selected, resulting in a total of 405 radiographs for analysis. To standardize the dataset, the images were cropped so that only the crown and root portions of the teeth were visible, while other anatomical structures were excluded.

Following preprocessing, each radiograph was divided into 15 to 100 segments. From these, one representative segment per radiograph was selected and used for further analysis. This procedure ensured that each radiograph contributed a single, standardized segment to the dataset.

This segmentation approach provided smaller, homogeneous image regions, facilitating more effective feature extraction and improving the learning capacity of the AI models.

#### Image Data Representation Method

Each segment was represented using the image data representation method, producing a set of numerical descriptors. 18 features included: Width, Height, Aspect Ratio, Brightness, Contrast, Entropy, Contrast-Weighted Entropy, Sharpness, Colorfulness, Edge Density, Saturation, Hue Variance, Texture Contrast, Texture Homogeneity, Histogram Skewness, Histogram Kurtosis, Histogram Peak and Dominant R/G/B values.

During the initial feature extraction phase, an automated feature generation tool was used, which produced a broad set of candidate variables including both grayscale- and color-oriented descriptors. However, preliminary analysis showed that the values of Colourfulness, Saturation, and Hue Variance were consistently zero across all segments, confirming that these features had no meaningful variability due to the grayscale nature of the panoramic radiographs. Therefore, although initially extracted, all color-based features were removed during preprocessing and excluded from model training. Similarly, the width and height parameters were discarded after verifying that all segments were generated with identical dimensions, making these attributes non-informative for classification.

The detailed definitions and formulas of the utilized features are provided in Table 1 (Image Feature Definitions). This numerical representation ensured a comprehensive characterization of each image segment, enabling meaningful comparison and supporting subsequent machine learning analysis.

### 2.3. Machine Learning Algorithms

The segmented radiographs, represented by numerical features, were analyzed using a range of machine learning algorithms in order to compare classification performance. A total of 11 algorithms were tested, which are presented in Table 2 (Machine Learning Algorithms).

Each algorithm was trained and tested using the segmented radiographs, grouped into the three caries categories. Performance metrics were calculated by varying the train-test ratios and the results were compared to determine which models provided the highest accuracy and reliability.

## 3. Results

In this study, a total of 1084 panoramic radiographs were initially examined. For balanced analysis, 135 radiographs were selected from each group (interproximal, occlusal and secondary caries), resulting in 405 radiographs in total. Each selected radiograph was divided into 15–100 image segments and one representative segment per radiograph was chosen for further analysis. Data was extracted using the image data representation method, resulting in numerical descriptors.

**Figure 1:**
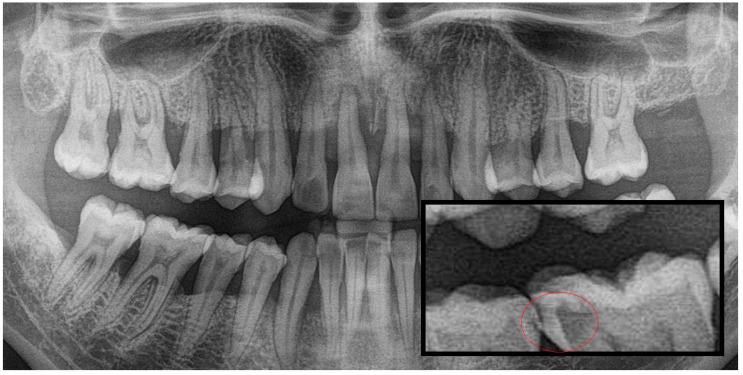
Panoramic radiography and segmented interproximal caries, red circle shows the caries.

**Figure 2:**
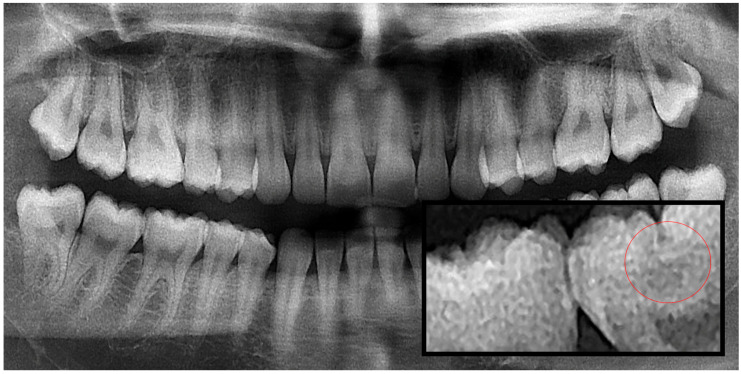
Panoramic radiography and segmented occlusal caries, red circle shows the caries.

**Figure 3:**
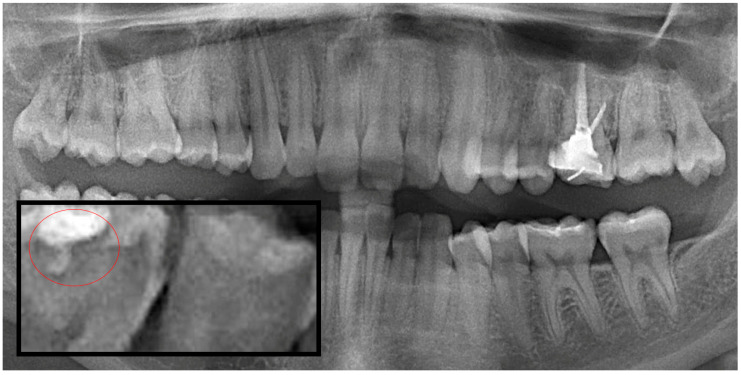
Panoramic radiography and segmented secondary caries, red circle show the caries.

Based on the data, the images were classified into three caries categories: Group 1 (interproximal caries), Group 2 (occlusal caries) and Group 3 (secondary caries). A variety of machine learning and neural network algorithms were applied, including Logistic Regression, Decision Tree, Gaussian Naïve Bayes (GaussianNB), Support Vector Machine (SVM), K-Nearest Neighbors (KNN), Random Forest, Bagged Trees, AdaBoost Classifier, Backpropagation Neural Network (BPNN), K-Means clustering and Radial Basis Function Neural Network (RBFNN) (Figure 1, Figure 2 and Figure 3).

**Figure 4:**
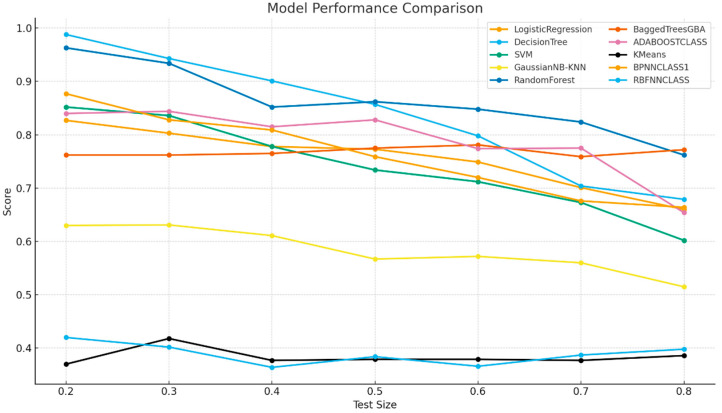
Model Performance Comparison.

**Figure 5:**
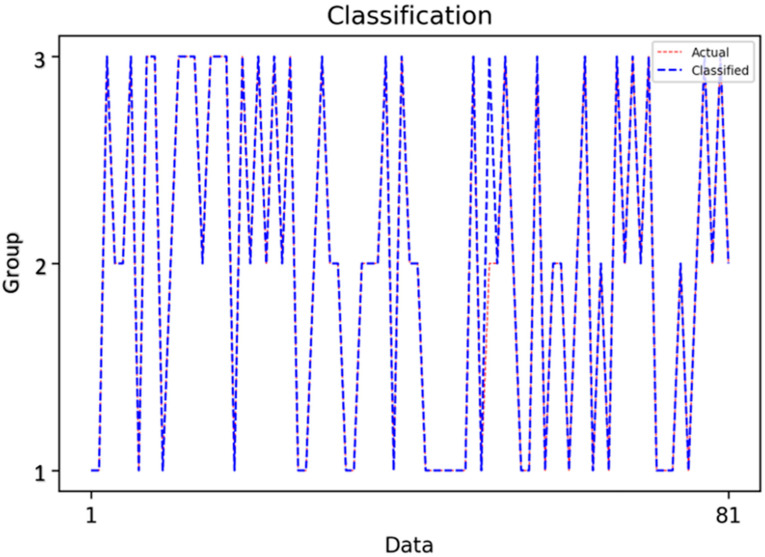
Classification Results of the Decision Tree Model.

The overall performance of the algorithms across test size or training proportion is illustrated in Figure 4. Among all tested algorithms, the Decision Tree model achieved the highest overall accuracy of 0.988 at the 0.2 train-test ratio, followed by Random Forest (0.963). Logistic Regression and SVM demonstrated moderate accuracy values (0.827 and 0.852 at 0.2, respectively), while Bagged Trees and AdaBoost yielded intermediate results (0.762–0.840). BPNN performed reasonably well at low train-test ratios (0.877 at 0.2) but declined with higher train-test ratios, whereas RBFNN consistently underperformed (0.364–0.420). GaussianNB and KNN remained low across all train-test ratios (~0.63) and K-Means clustering, as an unsupervised method, yielded the lowest accuracies (0.370–0.418) (Figure 5).

This figure demonstrates the classification outcomes of the Decision Tree model, highlighting the close alignment between actual and predicted class labels. At the 0.2 train-test ratio, the Decision Tree achieved near-perfect classification across the three caries categories, confirming its ability to generalize well when sufficient training data are available.

**Figure 6:**
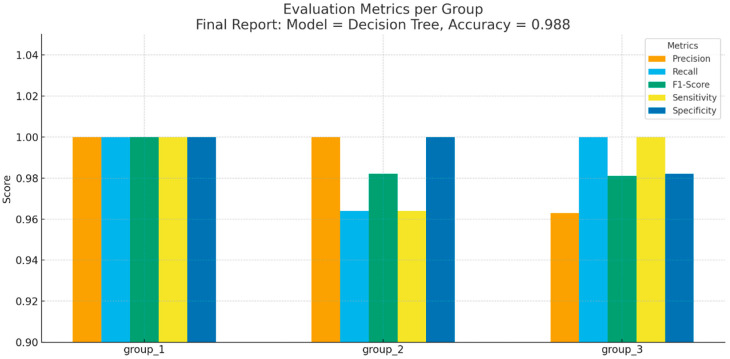
Evaluation Metrics per Group.

Detailed performance metrics for each class are presented in Figure 6.

- Group 1 (interproximal caries): All evaluation measures (Precision, Recall, F1-Score, Sensitivity, Specificity) reached 1.000, indicating perfect classification.
- Group 2 (occlusal caries): Precision = 1.000, Recall = 0.964, F1-Score = 0.982, Sensitivity = 0.964, Specificity = 1.000.
- Group 3 (secondary caries): Precision = 0.963, Recall = 1.000, F1-Score = 0.981, Sensitivity = 1.000, Specificity = 0.982.

These results show that the Decision Tree achieved not only high overall accuracy but also consistent performance across all caries subtypes.

### Ratio-Based and Methodological Discussion

At the 0.2 train-test ratio, Decision Tree delivered the highest accuracy (0.988), closely followed by Random Forest (0.963), reflecting the strength of tree-based models when ample training data are available. At the 0.8 train-test ratio, performance declined for all classifiers, but Random Forest (0.762) outperformed Decision Tree (0.679), underscoring the advantage of ensemble approaches in limited-data scenarios. Linear models such as Logistic Regression and SVM showed sharper declines, emphasizing their reliance on larger datasets for stable generalization.

Tree-based algorithms thus proved most effective, capturing the non-linear and hierarchical relationships inherent in the extracted features. Decision Tree combined superior accuracy with interpretability, making it particularly relevant for clinical decision support. Neural networks such as BPNN were competitive at lower train-test ratios but unstable at higher train-test ratios, while GaussianNB, KNN and K-Means were consistently inadequate for this task.

## 4. Discussion

The present study demonstrated that structured segmentation and feature extraction from panoramic radiographs can deliver extremely high diagnostic performance. By segmenting 1084 radiographs into 15–100 regions and transforming them using the image data representation method to generate numerical descriptors, our pipeline enabled multi-class classification of interproximal, occlusal and secondary caries. Among all tested algorithms, Decision Tree achieved the best performance (accuracy = 0.988 at 0.2 train-test ratio) with perfect classification of interproximal lesions and near-perfect outcomes for occlusal and secondary categories. Random Forest outperformed in higher train-test ratio scenarios, underscoring its robustness with reduced training data.

When contrasted with prior panoramic-based investigations, several clear distinctions emerge:

Oztekin et al. [27] trained CNN models (ResNet-50, EfficientNet-B0, DenseNet-121) on tooth-level panoramic crops extracted from 562 panoramic radiographs, reporting ~92% accuracy (sensitivity 87.33%, specificity 96.0%, F1 = 91.61%). While effective in binary classification of carious vs. non-carious teeth, their framework did not differentiate lesion subtypes or restoration-related conditions. In comparison, our structured feature approach not only yielded higher overall accuracy but also enabled granular multi-class classification, offering richer diagnostic detail.

Lin et al. [28] proposed lightweight CNNs for panoramic tooth segmentation, achieving IoU = 0.804 and Dice = 0.89 with minimal computational cost (~0.33 M parameters). Their experiments were conducted on 1321 panoramic radiographs, focusing on segmentation efficiency rather than diagnostic classification. In contrast, our pipeline demonstrated that segmented regions can serve as a foundation for accurate subtype-aware diagnostic classification, extending segmentation into clinically actionable outcomes.

Alharbi et al. [29] employed nested U-Nets (U-Net, U-Net++, U-Net3+) for cavity detection on a dataset of 1500 panoramic radiographs, achieving ~95% accuracy and strong pixel-level delineation (Dice ~0.84–0.90). Their models excelled at localization, but the task was restricted to cavity vs. no cavity. Unlike their binary lesion-level analysis, our study classified interproximal, occlusal and secondary caries with near-perfect accuracy, highlighting the added clinical value of numerical descriptors for subtype-specific classification.

Kwiatek et al. [30] clinically compared the Diagnocat AI platform with dentists on panoramic radiographs, explicitly differentiating primary vs. secondary caries. Their results showed higher agreement in molars and incisors, lower in premolars and variable performance in occlusal, labial and lingual surfaces. While their real-world validation underscores the challenges of clinical heterogeneity, diagnostic accuracies were lower and more inconsistent compared to our controlled dataset. Our pipeline consistently achieved near-perfect classification across all lesion categories, but further validation under real-world conditions will be necessary to confirm this robustness.

Lee et al. [31] investigated CNNs (Inception v3) on periapical radiographs of premolars and molars, achieving 89% and 88% accuracy with AUC up to 0.917. Their end-to-end CNN pipeline demonstrated expert-level performance but was limited to periapical radiographs and binary detection. By contrast, our panoramic-based pipeline reached higher accuracy and enabled multi-class subtype differentiation, while maintaining interpretability through Decision Tree and Random Forest models.

Taken together, these comparisons suggest that while CNN- and U-Net–based pipelines excel in localization and segmentation, they often remain limited to binary or lesion-level classification. Clinical benchmarking studies, such as Kwiatek et al., expose variability but report lower and heterogeneous accuracies. Our explicit segmentation–feature extraction approach delivered superior accuracy, interpretability and subtype-aware diagnostic granularity. The robustness of Decision Tree at low train-test ratios and Random Forest at high train-test ratios further illustrates the adaptability of tree-based methods. Nonetheless, broader external validation, multimodal imaging integration (panoramic + bitewing) and hybrid models that combine structured features with deep learning are needed to ensure generalizability and clinical adoption. Additionally, evaluating model performance across different demographic groups and imaging devices will further clarify the clinical scalability of the proposed framework.

Notably, while ensemble methods such as Random Forest showed competitive performance—especially in scenarios with limited training data (e.g., 0.8 train-test ratio)—they did not surpass the simplicity and clarity of the Decision Tree in full-data settings. In contrast, algorithms such as GaussianNB, KNN, RBFNN and K-Means clustering yielded significantly lower accuracies, indicating limited applicability for this specific problem and feature set. This performance disparity underscores the importance of choosing algorithms aligned with the mathematical properties of the extracted numerical descriptors.

Among the evaluated models, Decision Tree algorithm demonstrated near-perfect diagnostic performance, emphasizing their interpretability and practical clinical applicability. These results suggest that AI-assisted systems leveraging quantitative imaging data can function as reliable diagnostic support tools, enabling more consistent, timely and effective treatment planning.

The results of this study suggest that Decision Tree-based approaches, when paired with well-engineered numerical features from segmented panoramic radiographs, strike an optimal balance between predictive accuracy and clinical interpretability. Such models may serve as effective decision-support tools in dentistry, reducing subjectivity and accelerating diagnostic workflows. Moreover, the transparency of Decision Tree pathways aligns well with clinical expectations for explainable AI, facilitating clinician trust and adoption.

In this study, the integration of image segmentation, feature extraction and a diverse set of classification algorithms has demonstrated the strong potential of artificial intelligence for dental caries detection on panoramic radiographs. Among the eleven models tested, the Decision Tree achieved the highest accuracy (0.988 at the 0.2 train-test ratio), with perfect classification in Group 1 and near-perfect performance in Group 2 and Group 3. The robust performance across all caries subtypes confirms the method’s reliability and interpretability. The results of this methodological framework are of considerable clinical importance: more precise diagnosis and treatment planning not only improve patient outcomes but also enhance clinical efficiency. These findings highlight that structured numerical descriptors extracted from well-segmented regions can be as diagnostically informative as features learned by more complex deep models, particularly in data-limited contexts.

Based on the experimental results, H01 and H02 were confirmed, as both the image processing supported workflow and the machine learning algorithms demonstrated high diagnostic performance in detecting dental caries. However, H03 was partially accepted, because the models showed measurable differences in accuracy and sensitivity across interproximal, occlusal and secondary caries, indicating that performance was not uniform across all subtypes.

However, certain limitations must be acknowledged. This work is based on a single institutional dataset; in future studies external validation across multiple centers and imaging systems can be included in the study to confirm generalizability. In addition, while artifact-laden or low-quality images were excluded, real-world settings will include such challenging cases, which may reduce performance. Future versions of the system should therefore incorporate automated quality-control modules or preprocessing strategies to manage such variability.

Future studies will focus on expanding the dataset, optimizing feature extraction, and applying systematic hyperparameter tuning to both supervised and unsupervised algorithms. In particular, the K-Means clustering approach will be revisited with improved parameter settings to achieve more reliable clustering performance. Alternative clustering and classification methods will also be explored to enhance overall model robustness. Nonetheless, challenges such as dataset standardization, labeling consistency, privacy and clinical validation remain critical issues to be addressed in future research. Future studies should aim to incorporate cross-validation strategies, larger and more heterogeneous datasets and possibly hybrid models combining image-based features with patient level clinical data. Furthermore, evaluating longitudinal diagnostic consistency and integrating clinician feedback loops may strengthen real-world utility and support regulatory approval processes. The current study relied on a single institutional panoramic dataset; therefore, future work will include testing on external datasets and additional modalities such as bitewing and periapical radiographs. With the expansion of the dataset through multi-institutional collaborations, the proposed method will be evaluated using both machine learning and deep learning models to assess its generalizability. Ultimately, the integration of such technologies into dental practice holds great promise for advancing preventive care and improving overall oral health outcomes.

## 5. Conclusions

Building on previous research, our study obtained encouraging results, demonstrating that, for the first time, integrating numerical data from segmented panoramic images to artificial intelligence system improved the diagnostic accuracy of detecting dental caries in panoramic radiographs. By extracting and analyzing numerical image features, Decision Tree models in particular achieved near-perfect diagnostic performance, underscoring their clinical interpretability and practical value. These results indicate that AI-assisted systems can serve as reliable diagnostic support tools, minimizing subjectivity and facilitating more consistent, timely and effective treatment planning. The integration of such technologies into dental practice holds promise for advancing preventive care and improving overall oral health outcomes. Integrating advanced image processing techniques into everyday dental practice can lead to quicker and more efficient treatments, helping to lower the number of severe caries cases and enhance overall oral health. As these systems mature and are validated clinically, they hold promise to become integral components of dental imaging workflows, supporting clinicians with objective, consistent and rapid diagnostic insight.

## Figures and Tables

**Table 1:** Image Feature Definitions [20,21,22,23,24].

Feature Name	Description	Formula
Width	Horizontal dimension of an image in pixels (left to right). Used in resolution and aspect ratio calculations.	image.shape[1]
Height	Vertical dimension of an image in pixels (top to bottom). Defines resolution with width.	image.shape[0]
Aspect Ratio	Ratio between width and height (e.g., 16:9, 4:3, 1:1). Describes the overall shape of the image.	width ÷ height
Brightness	Average pixel intensity; low = dark image, high = overexposed.	(1/N) Σ I_i_
Contrast	Variation between light and dark areas; affects detail clarity.	√(1/N Σ (I_i_ − μ)^2^)
Entropy	A measure of randomness or variability in the data; low values indicate homogeneity; high values indicate complexity.	−Σ p_i_ log_2_(p_i_)
Contrast-Weighted Entropy	It is an image-processing metric that combines image contrast and entropy to evaluate an images overall quality or information richness.	CWE = C × H
Sharpness	Edge clarity and detail; used for focus and blur assessment.	Var(Δ^2^I)
Colorfulness	How rich and vibrant the colors are; colorful images appear lively.	√(σ_r_g^2^ + σ_γ_b^2^) + 0.3√(μ_r_g^2^ + μ_γ_b^2^)
Edge Density	Ratio of edge pixels to total pixels; indicates visual complexity.	(# edge pixels)/(# total pixels)
Saturation	Average color intensity; higher = more vivid, lower = more faded.	(1/N) Σ S_i_
Hue Variance	Variation in color tones; low in uniform scenes, high in colorful scenes.	(1/N) Σ (H_i_ − Ĥ)^2^
Texture Contrast	Gray-level variation within texture; used in material and surface analysis.	Σ (I − j)^2^ P(i,j)
Texture Homogeneity	Measures uniformity of texture; higher = smoother and less varied.	∑(i = 0 to N − 1) ∑(j = 0 to N − 1) P(i,j)/(1 + |i − j|)
Histogram Skewness	Asymmetry of intensity histogram; positive = dark-dominant, negative = bright-dominant.	(1/N) Σ ((I_i_ − μ)^3^/σ^3^)
Histogram Kurtosis	Peakedness of intensity histogram; high = sharp peaks and heavy tails.	(1/N) Σ ((I_i_ − μ)^4^/σ^4^)
Histogram Peak	Most frequent brightness level in the histogram; shows dominant intensity.	Maximum frequency in histogram.
Dominant R/G/B	Mode values of red, green and blue channels; represent overall image color tone.	Mode of each color channel histogram

**Table 2:** Machine Learning Algorithms [24,25,26].

Feature Name	Description
Logistic Regression (LR)	Logistic Regression predicts categorical outcomes using a sigmoid transformation of input features. It is interpretable, efficient for linearly separable data and benefits from regularization to prevent overfitting.
Decision Tree (DT)	Decision Trees divide datasets into hierarchical branches based on information gain or Gini impurity. They provide transparent, rule-based decisions but tend to overfit on small or noisy datasets.
Support Vector Machine (SVM)	SVM constructs an optimal boundary that maximizes the margin between different classes. By applying kernel functions, it can efficiently handle nonlinear and high-dimensional data.
Gaussian Naïve Bayes (GNB)	Naïve Bayes uses probabilistic reasoning based on Bayes’ theorem, assuming independence among features. The Gaussian version is suitable for continuous data and performs well even with limited training samples.
K-Nearest Neighbors (KNN)	KNN classifies new samples according to the majority label among their nearest neighbors in the feature space. It is simple and effective but becomes computationally expensive for large datasets.
Random Forest (RF)	Random Forest combines multiple decision trees trained on random subsets of data and features. It enhances model robustness, reduces overfitting and delivers high classification accuracy.
Bagged Trees (Bagging)	Bagging creates multiple models from bootstrapped samples and aggregates their predictions to lower variance. This ensemble approach improves stability and reduces overfitting in decision-tree-based methods.
AdaBoost Classifier	AdaBoost builds an ensemble of weak classifiers, iteratively focusing on examples that were previously misclassified. It increases overall accuracy but can be sensitive to noise in the dataset.
K-Means Clustering	K-Means is an unsupervised algorithm that organizes data into a predefined number of clusters by minimizing within-cluster distances. It is computationally efficient but sensitive to initial centroid placement.
Backpropagation Neural Network (BPNN)	BPNN is a multilayer neural network that learns by propagating output errors backward to adjust connection weights. It is powerful for modeling nonlinear relationships but requires careful tuning and sufficient data.
Radial Basis Function Neural Network (RBFNN)	RBFNN uses radial basis functions (such as Gaussian) in its hidden layer to approximate nonlinear mappings. It trains faster than multilayer perceptrons and provides smooth function approximation.

## Data Availability

The data supporting the outcomes of this study can be provided by the corresponding author upon reasonable request. The data are not publicly available due to privacy or ethical restrictions and the authors have chosen not to share their data.

## Abbreviations

The following abbreviations are used in this manuscript:

AI	Artificial Intelligence
ML	Machine Learning
DL	Deep Learning
CNN	Convolutional Neural Network
BPNN	Backpropagation Neural Network
RBFNN	Radial Basis Function Neural Network
SVM	Support Vector Machine
KNN	K-Nearest Neighbors
NB/ GaussianNB	Naïve Bayes/ Gaussian Naïve Bayes
DT	Decision Tree
RC	Random Forest
AUC	Area Under the Curve
F1	F1-Score (harmonic mean of precision and recall)
OPG	Orthopantomograph (Panoramic Radiograph)
ROI	Region of Interest
